# Receptor complexes for each of the Class 3 Semaphorins

**DOI:** 10.3389/fncel.2012.00028

**Published:** 2012-07-05

**Authors:** Anil Sharma, Joost Verhaagen, Alan R. Harvey

**Affiliations:** ^1^School of Anatomy, Physiology and Human Biology, The University of Western Australia, CrawleyWA, Australia; ^2^Netherlands Institute for Brain Research, MeibergdreefAmsterdam, Netherlands

**Keywords:** adhesion molecules, axon guidance, axon repulsion, Neuropilins, Plexins, Robo, Semaphorins

## Abstract

The Class 3 Semaphorins (Sema3s) are a sub-family of proteins whose known biological roles are varied and growing. The mechanism of action of the Sema3s requires binding to transmembrane receptors that comprise heteromeric complexes of Neuropilins, Plexins and cell adhesion molecules (CAMs). However, knowledge of the receptor components of the Sema3s remains incomplete, and there may be receptor components which are as yet undiscovered. The receptor complexes of the Sema3s share receptor components with each other, and it is the specific combination of these components within a heteromeric complex that is thought to give rise to selective binding and signalling for individual Sema3s. This crosstalk makes it experimentally difficult to define a single holoreceptor for each Sema3. Furthermore, the receptor composition for a given Sema3 may differ between cell types, and change as a function of developmental state or pathological situation. Nevertheless, there are at least some known differences in the constitutive structure of the receptors for the Sema3s. For example in neural cells, *Sema3a* and *Sema3f* signal through different Neuropilins (*Nrp1* and *Nrp2* respectively) and *L1cam* only appears important for Sema3a signaling, while *Nrcam* forms a complex with *Nrp2*. Further complexity arises from crosstalk of other families of ligands (e.g., *VEGF*) with Sema3 receptor components. Thus the Sema3s, which have been shown as antagonists for each other, can also act as antagonists for other families of molecules. This review compiles experimental evidence describing the receptor components for the Sema3s, detailing the current state of knowledge of which components are important for signaling of each Sema3 before going on to consider possible future directions for the field.

## Introduction

The Class 3 Semaphorins (Sema3s) were first discovered as axon guidance molecules (Kolodkin et al., 1992; Luo et al., 1993), and in vertebrates are the only secreted members of the Semaphorin family (Semaphorin Nomenclature Committee, 1999). The known Sema3s consist of *Sema3a* through *Sema3g* (Kolodkin et al., 1993; Luo et al., 1993, 1995; Püschel et al., 1995; Roche et al., 1996; Sekido et al., 1996; Xiang et al., 1996; Feiner et al., 1997; Stevens and Halloran, 2005; Taniguchi et al., 2005), and their known physiological and pathological functions have expanded to include axon attraction and repulsion, apoptosis, cell migration, growth cone collapse, immune response, organogenesis, tumour suppression and promotion, and vasculature development (Yazdani and Terman, 2006; Roth et al., 2009; Takegahara and Kumanogoh, 2010; Staton, 2011; Sakurai et al., 2012; Takamatsu and Kumanogoh, 2012). Vital to our understanding of these functions of the Sema3s is our understanding of their receptors. However, our knowledge of the composition of the holoreceptors for the Sema3s is far from complete (Raper, 2000), and we lack a recent detailed and comprehensive review of what is known.

In this review we present the current state of knowledge on Sema3 receptors, deduced from bioassays and biochemical and *in vivo* analyses, with particular emphasis on neural cells. It is our hope that this review helps to shed light on those areas most in need of further research.

## General structure of a class 3 semaphorin receptor

The receptors for the Sema3s are heterocomplexes of receptor subunits, with significant overlap between different Sema3 holoreceptors (Feiner et al., 1997; Takahashi et al., 1998; Rohm et al., 2000). The molecules first identified as receptors for the Sema3s were Neuropilin 1 and 2 (*Nrp1*, *Nrp2*), independently reported by two laboratories in 1997 (Chen et al., 1997; He and Tessier-Lavigne, 1997; Kolodkin et al., 1997). Both *Nrp1* and *Nrp2* were found to be essential for Sema3 signal transduction, but the specificity of Sema3 signaling could not be attributed to either Neuropilin alone (Chen et al., 1997; Takahashi et al., 1998). Indeed, even before the discovery that the Neuropilins were essential for Sema3 signaling, it was inferred from the structure of *Nrp1* and its expression profile in the developing mouse nervous system that *Nrp1* was likely heterophilic (Kawakami et al., 1996). Also, the cytoplasmic domains of *Nrp1* were found to be not required for Sema3 signaling (Nakamura et al., 1998; Renzi et al., 1999), indicating that Neuropilins act in concert with other receptor co-receptors which transduce the extracellular signals to the intracellular signaling pathways.

Class A Plexins (PlexinAs) are the main co-receptors for the Sema3s, and were the first identified (Takahashi et al., 1999; Tamagnone et al., 1999). These initial studies found that PlexinAs associate with the Neuropilins and that this association is important for signal transduction of the Sema3s. Further studies discovered a number of other co-receptors for the Sema3s: *L1cam* (Castellani et al., 2000), *Nrcam* (Falk et al., 2005), *Plxnb1* (Usui et al., 2003), and *Plxnd1* (Gitler et al., 2004; Gu et al., 2005; Chauvet et al., 2007). A summary of known receptor components for each of the Sema3s is presented in Table 1.

**Table 1:** **Known receptor-ligand interactions**.

	***Sema3a***	***Sema3b***	***Sema3c***	***Sema3e***	***Sema3f***	***Sema3g***
*Nrp1*	+	+	+	+/−	+/−	−
*Nrp2*	−	+	+	−	+	+
*Plxna1*	+/−	?	+/−	?	+	?
*Plxna2*	+/−	?	+	?	+	?
*Plxna3*	+/−	?	?	?	+/−	?
*Plxna4*	+	?	?	?	+/−	?
*Plxnb1*	+	?	+	?	?	?
*Plxnd1*	+/−	?	+	+	?	?
*L1cam*	+	−	?	−	?	?
*Nrcam*	?	+	?	?	+	?
*Robo1*	?	?	?	?	?	?
*Chl1*	+/−	?	?	?	?	?

*+ receptor/co-receptor necessary for signal transduction; – receptor/co-receptor not necessary for signal transduction; +/– receptor/co-receptor necessary for signal transduction under some circumstances; ? no evidence on requirement of receptor/co-receptor for signal transduction*.

Reviews of the general structure and signaling of the Sema3 holoreceptors have been published previously (Pasterkamp and Kolodkin, 2003; Geretti et al., 2008; Pellet-Many et al., 2008; Zhou et al., 2008; Yoshida, 2012), consequently these concepts are only briefly revisited here. With the exception of *Sema3e*, all Sema3 receptors require a Neuropilin to act as the binding site for the Sema3 ligand. The binding of the Sema3 ligands to the Neuropilins depends on their N-terminus Sema sequence, and a 70 amino acid stretch within that sequence determines specificity (Koppel et al., 1997). The receptors for the Sema3s are multimeric, with varying numbers of associated Plexins or cell adhesion molecules (CAMs) providing the intracellular signaling mechanics. *Sema3e* differs in that it is able to bind directly to *Plxnd1* in the absence of a Neuropilin (Gu et al., 2005). The Plexins (Class A, with the exception of *Plxnd1* for *Sema3e*) and CAMs (*L1cam* and *Nrcam*) *cis*-interact with *Nrp1* and *Nrp2* through their transmembrane, and extracellular domains (Tamagnone et al., 1999; Rohm et al., 2000; Takahashi and Strittmatter, 2001; Castellani et al., 2002; Roth et al., 2008).

## Methodologies used to investigate receptor compositions

**Figure 1:**
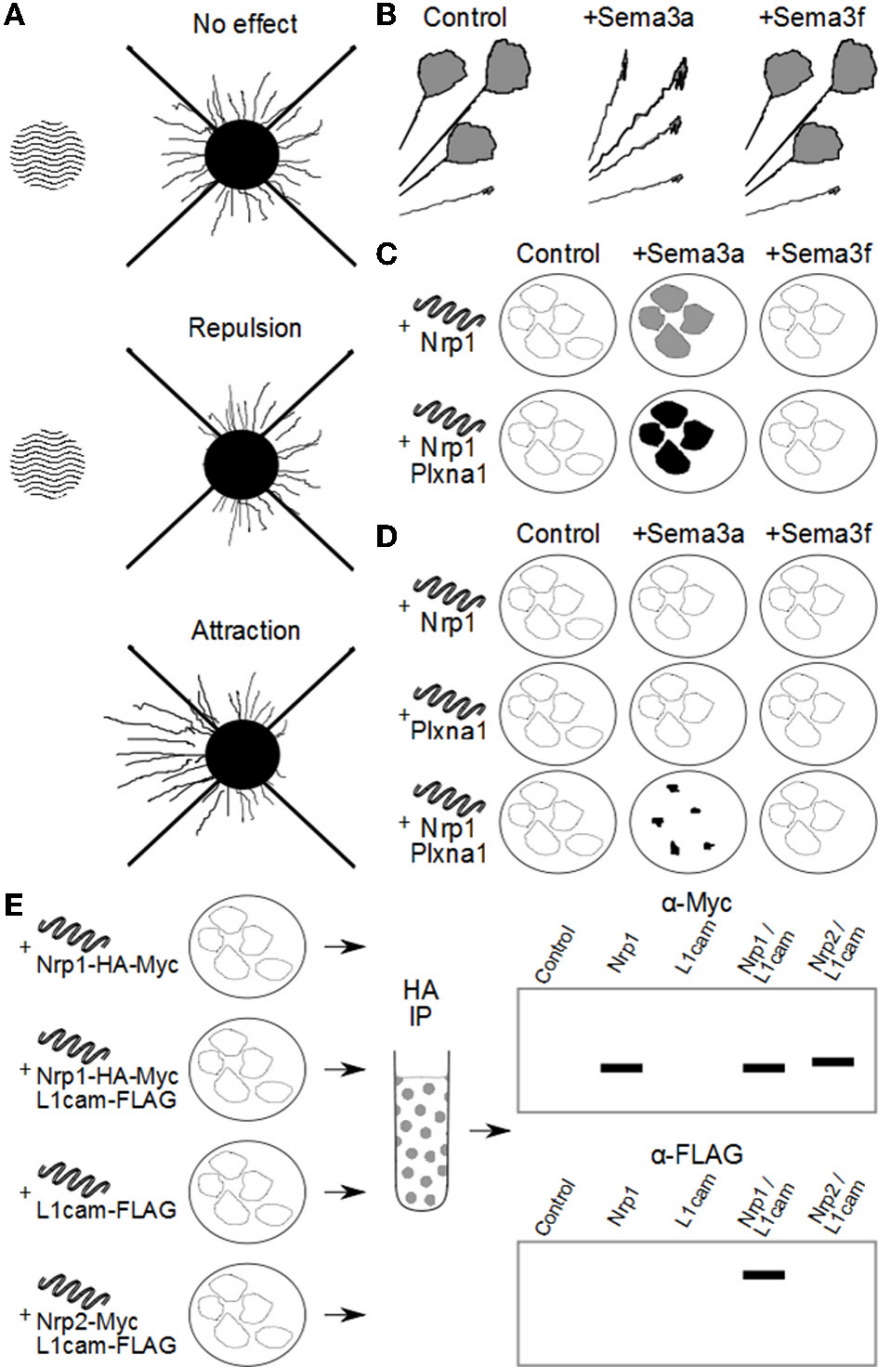
**Methods used to investigate Sema3 receptor components. (A)** Attraction/repulsion assay. The area of wavy lines represents aggregates of COS/HEK-293 cells over-expressing a Sema3. To the right is neuronal tissue with outgrowth of neurites. In this assay, the average or total lengths of neurites in the proximal and distal quadrants of the neuronal tissue (with respect to the COS/HEK-293 cell aggregates) are compared to quantify the amount of attraction or repulsion. **(B)** Neurites *in vitro* with active growth cones are exposed to exogenous Sema3 or vehicle. Changes in the percentage of active to collapsed growth cones are used to determine the biological activity of the Sema3s on different neuronal types. In this example, neurites are found to collapse in the presence of *Sema3a* but not *Sema3f*. **(C)** COS/HEK-293 cells are transfected to ectopically express one or more putative Sema3 receptor components. These cells are then exposed to exogenous recombinant Sema3 which includes an artificial conjugate, most often alkaline phosphatase. After incubation at 4°C the cultures are washed and stained either by immunocytochemistry, or direct application of a chromagen that interacts with the artificial conjugate (for example NBT/BCIP with alkaline phosphatase). The intensity of staining is proportional to the binding kinetics of the recombinant Sema3 to the putative receptor components and/or complexes, and the dissociation constant can be derived by Scatchard analysis. In this way researchers can compare the relative affinity for the Sema3s to putative receptor components and complexes. For example, in this diagram, *Sema3a*, but not *Sema3f* binds to cells expressing *Nrp1*, and *Sema3a* binds more to cells expressing both *Nrp1* and *Plxna1*, than *Nrp1* alone. **(D)** COS cells engineered to over-express one or more putative Sema3 receptor components collapse on exposure to exogenous Sema3, if those receptor components form a functional receptor for that Sema3. In the example here, *Nrp1* or *Plxna1* can transduce a *Sema3a* signal when expressed in concert, but not individually. Furthermore, *Nrp1* and *Plxna1* cannot transduce a *Sema3f* signal, either individually or together. **(E)** Co-immunoprecipitation experiments allow researchers to investigate whether two or more receptor components interact either *in vivo*, or *in vitro*. In this example HEK-293 cells are engineered to ectopically express combinations of either *Nrp1* or *Nrp2*, and *L1cam*. The recombinant proteins are conjugated to artificial epitopes which allows for their selective immunoprecipitation, in this case by the HA tag on the *Nrp1*/*Nrp2*. Once selected for, the proteins are analysed by western blot for any other proteins of interest that were bound to *Nrp1*/*Nrp2* in the cell. In this example *L1cam* is analysed by the immunoblot, and appears associated with only *Nrp1*, and not *Nrp2*. Importantly the controls reveal that the *L1cam* signal is not detected in cells expressing *L1cam* alone, confirming the positive signal in *Nrp1*/*L1cam* cultures as due to *Nrp1* and *L1cam* interaction.

A number of *in vitro* methods have been used to investigate the function of the Sema3s: axon repulsion/attraction assays, grown cone collapse assays, COS cell collapse, co-immunoprecipitation, and ligand binding assays. To aid the reader in understanding how deductions about the makeup of Sema3 receptor complexes have been made, an overview of the methods used to elucidate the makeup of the Sema3 receptor complexes is given in Figure 1, and a brief description of these assays is given below.

Having first been discovered as repulsive axon guidance molecules, it was natural for investigators to use this function to dissect the receptor makeup for the Sema3s. Neural tissue explants [usually embryonic dorsal root glia (DRG)] are grown in a collagen matrix adjacent to cells (normally HEK-293 or COS cells) transfected to over-express a specific Sema3. The collagen matrix allows the formation of a gradient of Sema3s from the transfected cells toward the DRG explants. The most common method for quantifying the repulsion or attraction this bioassay is by measuring the length of neurites exiting the explant in the quadrants proximal and distal to the Sema3 source. Average neurite lengths are then either compared directly, or represented as a ratio of proximal/distal neurite lengths (Messersmith et al., 1995; Kolodkin et al., 1997).

Another bioassay enabling analysis of neuronal response to Sema3s is a growth cone collapse assay. Explants are grown *in vitro* and then exposed to exogenous Sema3 ligand for up to one hour before fixation and staining. The number of collapsed versus non-collapsed growth cones is then counted and compared by statistical means (Kapfhammer et al., 2007). It is important to note that this method captures only inhibitory effects on growth cones, and may miss any attractant or growth promoting effects (Campbell et al., 2001).

Another technology that has proved useful for investigating the Sema3 receptors is to engineer COS and HEK-293 cells to over express different combinations of Sema3 receptor components. When COS cells express a minimum functional Sema3 receptor, they collapse if presented with that Sema3 (Takahashi et al., 1999), thus elucidating necessary components for each Sema3 receptor. Further, COS and HEK-293 cells that over express combinations of receptor components have allowed researchers to study the interactions of these components with each other by co-immunoprecipitation. This technique is especially important, as until recently reliable commercial antibodies against specific Sema3s and their receptor components were scarce, making co-immunoprecipitation from animal tissue difficult at best. Ectopic expression of Sema3 receptor subunits also allows measurement of binding affinities of the Sema3s to different permutations of the general Sema3 receptor complex. For example, recombinant Sema3 ligands fused to a reporter sequence (for example FLAG/*Myc*/alkaline phosphatase) are exposed to engineered COS/HEK-293 cells, level of binding then visualized via the reporter sequence, and binding affinities calculated by Scatchard analysis (Chen et al., 1997; He and Tessier-Lavigne, 1997).

Recombinant Sema3 ligands conjugated to reporter sequences have also been used to map binding to different tissues both *in vitro* and *ex vivo*. Analysis of the expression of the different Sema3 receptor constituents in different tissues has then allowed deduction of the necessity of individual receptor components in each Sema3 holoreceptor.

The above methods have been combined with various models to study the makeup of the Sema3 receptors: genomic knock-out/knock-in, gene knock-downs, protein over expression, functional blocking antibodies, and tissues that are known to express different combinations of the Sema3 receptor components (for example sympathetic neurons versus sensory neurons). In addition to the above, researchers have also carefully analysed the *in vivo* phenotypes of single and double knock-out mutant animals.

## Sema3a

*Sema3a* was the first Sema3 to be discovered (Kolodkin et al., 1992; Luo et al., 1993), and has been the most widely studied. As a result, knowledge of the *Sema3a* receptor is the most complete of all the Sema3s. Nonetheless, the make-up of the *Sema3a* receptor remains somewhat equivocal.

### Neuropilins

#### Nrp1

*Nrp1* was identified as a receptor for *Sema3a* by screening for *Sema3a*-AP binding of COS cells transfected with a cDNA expression library from E14 rat dorsal root ganglia (DRG, sensory neurons that bind recombinant *Sema3a*) (He and Tessier-Lavigne, 1997; Kolodkin et al., 1997). The same studies confirmed *Nrp1* as a candidate receptor protein for *Sema3a* by demonstrating that the proteins co-immunoprecipitate, and that *Sema3a*-AP binds to exogenously expressed recombinant *Nrp1* in COS cells.

Additional evidence for *Nrp1* as a receptor for *Sema3a* came from studies using functional blocking antibodies against *Nrp1*. Anti-*Nrp1* antibodies ablate the axon repulsion and growth cone collapse effect of recombinant *Sema3a* on E14 rat DRGs (He and Tessier-Lavigne, 1997; Kolodkin et al., 1997) and sympathetic neurons (Chen et al., 1998). Also, anti-*Nrp1* antibodies abolish *Sema3a* mediated axon repulsion of mouse cortical neurons (Castellani et al., 2000). Similarly, *Npr1^Sema^* knock-out (a targeted deletion in the Sema region of *Nrp1*, removing Sema3 binding capability but leaving *VEGF* binding intact) in mice, causes *Sema3a* insensitivity in embryonic DRG neurons (Kitsukawa et al., 1997; Gu et al., 2003).

Corresponding evidence that *Nrp1* is a receptor for *Sema3a* comes from over expression studies; *Sema3a* insensitive chick E8 retinal neurons become sensitive to *Sema3a* mediated growth cone collapse after they are transduced with recombinant *Nrp1* (Nakamura et al., 1998). Furthermore, in *Xenopus*, retinal neurons become responsive to *Sema3a* induced growth cone collapse during development, concomitant with expression of *Nrp1*, and earlier sensitivity can be conferred using transfection to ectopically express *Nrp1* (Campbell et al., 2001).

#### Nrp2

Initially it was shown that, unlike *Nrp1*, *Nrp2* is unable to bind *Sema3a in vitro* (Chen et al., 1997; Takahashi et al., 1998). Also, soluble *Nrp2*-Fc abolishes *Sema3c* and *Sema3f* induced axon repulsion of embryonic rat sympathetic neurons *in vitro*, but has no effect on *Sema3a* induced repulsion in the same model (Chen et al., 1998). However, more recently *Sema3a* has been found to bind to COS cells expressing *Nrp2* (albeit 1.3 fold less than *Nrp1* expressing COS cells), and a functional blocking antibody against *Nrp2* removes the chemorepulsive effect of *Sema3a* on at least one human glioma cell line *in vitro* (Nasarre et al., 2009). *In vivo*, *Sema3a* binding to tissue is abolished in areas such as the olfactory bulb in *Nrp1^Sema^* knockouts, but is still present in other tissues (Cariboni et al., 2011). Indeed, *Sema3a* binding is only completely absent in *Nrp1^Sema^*/*Nrp2* double knockouts, and both Neuropilins appear to be partially redundant for one another in transducing *Sema3a* signals in mouse vomeronasal axons (Cariboni et al., 2011). Thus while *Nrp1* appears to be an essential component of a functional *Sema3a* receptor complex, *Nrp2* may or may not be necessary, depending on the cell type.

### Class a plexins

COS cells ectopically expressing both *Nrp1* and *Plxna1* undergo collapse, or rounding up, in the presence of *Sema3a*, but no such reaction occurs if only *Nrp1* or *Plxna1* is expressed (Takahashi et al., 1999; Takahashi and Strittmatter, 2001). Moreover, the presence of *Plxna1* increases the affinity of *Nrp1* for *Sema3a in vitro*, and *Nrp1* and *Plxna1* cluster in E7 chick DRG growth cones in response to exogenous *Sema3a* (Takahashi et al., 1999). In addition, expression of a dominant negative *Plxna1* protein (lacking the intracellular domain) abolishes growth cone collapse in response to *Sema3a* in chick E7 DRG neurons (Takahashi et al., 1999), mouse E12.5 sensory ganglia (Rohm et al., 2000), and X*enopus laevis* spinal neurons (Tamagnone et al., 1999). Intriguingly, it has been suggested that the presence of *Plxna1* in the *Sema3a* holoreceptor acts as an adapter for the association of *Plxnb1*, increasing the possible signaling cascade complexity (Usui et al., 2003).

Similarly, embryonic mouse sensory ganglia lose their sensitivity to exogenous *Sema3a in vitro* when induced to express ectopic dominant negative *Plxna2* protein (Rohm et al., 2000). Furthermore, COS cells expressing both *Plxna2* and *Nrp1* collapse in the presence of *Sema3a*, but not if expressing only one of *Plxna2* or *Nrp1* (Takahashi and Strittmatter, 2001). Finally, co-expression of both *Plxna2* and *Nrp1* in HEK-293 cells increases their affinity for *Sema3a* above those expressing *Nrp1* alone (Rohm et al., 2000).

However, while both *Plxna1* and *Plxna2* are important for *Sema3a* signaling, neither *Plxna1* nor *Plxna2* appear to be essential components of the *Sema3a* holoreceptor. In mouse knockout models, the facial nerve of *Plxna1* and *Plxna2* knockouts does not exhibit the same level of abnormal phenotype as the *Sema3a* knockout (Schwarz et al., 2008). This indicates some degree of redundancy in the make-up of the full *Sema3a* receptor, with different components conveying overlapping signaling capabilities.

Such redundancy is particularly marked with *Plxna3* and *Plxna4* (Schwarz et al., 2008). *Plxna3* knockouts have reduced sensitivity to *Sema3a* mediated axon repulsion and growth cone collapse in mouse embryonic DRG, hippocampal neurons, and embryonic sympathetic neurons (Cheng et al., 2001; Bagri et al., 2003; Yaron et al., 2005). Similarly, *Plxna4* knockouts display a reduced sensitivity of mouse embryonic DRG and sympathetic neurons (Suto et al., 2005; Yaron et al., 2005). However, *Plxna3*/*Plxna4* double-knockouts completely abolish sensitivity to *Sema3a* induced growth cone collapse and axon repulsion (Yaron et al., 2005), strongly suggesting that *Plxna3* and *Plxna4* are partially redundant for each other within the *Sema3a* holoreceptor. Further evidence for the reciprocal redundancy of *Plxna3* and *Plxna4* in the *Sema3a* receptor is found in the migration of sympathetic neurons, which is disrupted by double, but not single, *Plxna3* and *Plxna4* knockouts (Waimey et al., 2008). Finally, *Plxna3* is required for *Sema3a* induced neuron cell death, but the absence of *Plxna4* also reduces this effect of *Sema3a* by around 50% (Ben-Zvi et al., 2008).

It appears from the above evidence that *Plxna3* and *Plxna4* are part of the *Sema3a* holoreceptor, but partially mutually redundant, probably sharing overlapping signaling mechanisms. Interestingly, co-expression of *Plxna3* and *Nrp1* is not sufficient to confer sensitivity of COS cells to *Sema3a* mediated cell collapse, (Takahashi and Strittmatter, 2001), again indicating that whilse *Plxna3* is part of the *Sema3a* holoreceptor, it is not essential. However it must be noted that all experimental avenues have as yet not been exhausted, as illustrated by a very recent report showing that *Plxna4* is essential for *Sema3a* signaling in HUVECs, an endothelial cell line (Kigel et al., 2011).

One other Plexin that has been studied as a putative component of the *Sema3a* receptor is *Plxnd1*. *Sema3a* binding to *Nrp1* is enhanced by the presence of *Plxnd1*, at least in transfected COS cells *in vitro* (Gitler et al., 2004), indicating a possible role for *Plxnd1* in *Sema3a* signaling. However, there have been no functional studies performed to test this hypothesis.

### L1cam

*L1cam* has been demonstrated to be important for signal transduction within the *Sema3a* receptor complex (Castellani et al., 2000, 2002, 2004). Mouse cortical and DRG neurites, normally repulsed by *Sema3a* in co-culture assays, are indifferent to *Sema3a* when *L1cam* is genetically knocked out (Castellani et al., 2000; Bechara et al., 2008). Importantly, COS cells over-expressing *L1cam* and *Nrp1* collapse in the presence of *Sema3a*, indicating that *L1cam* can transduce the *Sema3a* signal (Castellani et al., 2004).

Interestingly, *L1cam* appears to be important for *Nrp1*, but not *Nrp2*, signaling. When co-expressed in COS cells, both *Nrp1* and *L1cam*, but not *Nrp2* and *L1cam*, naturally associate, independent of the presence of *Sema3a* (Castellani et al., 2000). Furthermore, *Nrp1* and *L1cam* from postnatal mouse brain lysates also co-immunoprecipitate, with the extracellular portion of *L1cam* sufficient for binding of *L1cam* to *Nrp1* (Castellani et al., 2000).

Nevertheless, the presence of *L1cam* is not necessary for the binding of *Sema3a* to *Nrp1*, at least in COS cells *in vitro* (Castellani et al., 2000). Furthermore, *L1cam* is not a necessary component of the *Sema3a* receptor, as shown under a number of circumstances. For example, neuronal apoptosis induced by *Sema3a* signaling is not affected by the lack of functional *L1cam* (Ben-Zvi et al., 2008). Therefore, as with other co-receptors for *Sema3a*, *L1cam* can transduce *Sema3a* signals but it is not essential for all *Sema3a* signaling under all circumstances.

Finally, the effect of the presence of *L1cam* in the *Sema3a* receptor is more nuanced than simply enabling/disabling *Sema3a* signaling. *L1cam* dependent, *Sema3a* induced neurite repulsion *in vitro* is converted into attraction by the addition of L1-Fc, the extracellular domain of *L1cam* fused with and Fc immunoglobulin fragment (Castellani et al., 2000), and similar extracellular soluble *L1cam* is present *in vivo* (Maretzky et al., 2005). Thus the presence of *L1cam* in the *Sema3a* receptor complex enables the cell to respond to the levels of both *Sema3a* and soluble *L1cam*.

### Chl1

*Chl1* (Close homologue of L1) associates with *Nrp1* both *in vivo* and *in vitro*, and is necessary for *Sema3a* induced embryonic mouse thalamic and cortical neuron growth cone collapse (Wright et al., 2007; Schlatter et al., 2008). Interestingly, *Chl1* mediated *Sema3a* signaling is dependent on a juxtamembrane region of *Chl1'*s cytoplasmic domain, conserved with *L1cam* (Wright et al., 2007). However, the number of studies of *Chl1* and Sema3 signaling are limited, and it is not possible to say whether *Chl1* is an essential receptor component generally or only has a role in specific cell types.

### Robo1

To date there has been one report of *Robo1* acting as a co-receptor for *Sema3a* (Hernández-Miranda et al., 2011). Co-immunoprecipitation from embryonic mouse forebrain, and COS cells over-expressing *Nrp1, Nrp2, Plxna1, Plxna4*, and *Robo1* demonstrated that *Robo1* can form a complex with *Nrp1*, but not *Nrp2, Plxna1*, or *Plxna4* individually. Further evidence was gathered from a covasphere aggregation assay [immunofluorescent beads (green or red) coated with relevant Fc proteins; heterophilic aggregates are yellow, homophilic aggregates are green or red], where *Robo1* interacted with *Nrp1*, but not *Nrp2*, *Plxna1*, or *Plxna4*. Also, co-immunoprecipitation of *Robo1* with *Plxna1*, and *Plxna4* from embryonic mouse forebrain lysates indicates that it can bind to *Nrp1* in complex with *Plxna1* and *Plxna4*, a complex that would constitute a functional *Sema3a* receptor.

This binding of *Robo1* to *Nrp1* depends, at least in part, on the first two Ig domains in *Robo1*, but not the final three. Also, by using the same covasphere assay it was found that *Robo1* cannot bind *Sema3a* directly, and *Sema3a* did not bind to COS cells ectopically expressing *Robo1* alone. It is still unclear, however, whether *Robo1* is part of the *Sema3a* receptor complex. The chemorepulsive effects of *Sema3a* and *Sema3f* on both mouse embryonic medial ganglionic eminence GN11 cells were reduced in *Robo1* knockout animals, despite the fact that *Robo1* only interacts with *Nrp1* and not *Nrp2*, an essential component of the *Sema3f* receptor complex. Indeed it appears that the effect of *Robo1* knockout on *Sema3a* and *Sema3f* induced chemorepulsion is the concomitant down-regulation of *Nrp1* and *Plxna1* expression. The mechanism by which *Robo1* expression modulates *Nrp1* and *Plxna1* expression is as yet unknown, however, this genetic interaction is in itself an intriguing new facet of Sema3 receptor biology.

## Sema3b

Unlike *Sema3a*, *Sema3b* binds to both *Nrp1* and *Nrp2* following ectopic expression in COS cells *in vitro* (Takahashi et al., 1998). Soluble *Nrp2*-Fc binds to mouse sub-ventricular zone (SVZ) regions expressing *Sema3b*, and this binding is markedly reduced or extinguished in *Sema3b* knockouts (Falk et al., 2005). Both anti-*Nrp2* antibodies and exogenous soluble *Nrp2* (*Nrp2-Fc)* abolished *Sema3b* induced attraction of mouse neonatal cortical neurons (Falk et al., 2005). Also, chick embryonic DRG neurons that are normally insensitive to *Sema3b* induced growth cone collapse *in vitro* become sensitized upon transduction with recombinant *Nrp2* (Takahashi et al., 1998). While *Sema3b* can bind both *Nrp1* and *Nrp2*, and it is known that *Nrp2* is essential for *Sema3b* signal transduction, it remains unclear whether the presence of *Nrp1* is necessary even though *Sema3b* can bind to *Nrp1* in the absence of *Nrp2*, as demonstrated by its competitive antagonism to *Sema3a* in embryonic chick RGCs (which express *Nrp1* but not *Nrp2*) (Takahashi et al., 1998).

Similarly, there is no evidence yet for the Plexins as constituents of the *Sema3b* receptor complex. However several IgCAMs have been investigated, with *Nrcam* but not *Tag-1*, *Contactin*, or *L1cam* able to bind *Nrp2* when co-expressed in HEK-293 *in vitro* (Castellani et al., 2000; Falk et al., 2005). *L1cam* is not required for *Sema3b* signal transduction in neonatal mouse cortical neurons (Castellani et al., 2000), and *Nrcam*, either in its soluble or membrane bound form, cannot bind *Sema3b* directly (Falk et al., 2005) However, *Nrcam* is essential for neonatal mouse cortical neuron sensitivity to *Sema3b* (Falk et al., 2005). Further, functional blocking antibody against *Nrcam* abolishes *Sema3b* mediated growth cone collapse and axon attraction of neonatal mouse cortical neurons (Falk et al., 2005). Intriguingly, *Nrcam* can mediate neuronal sensitivity to *Sema3b* by reducing *Calpain1* proteolytic activity on *Plxna1* (Nawabi et al., 2010).

Lastly, there is no direct evidence for *Robo1* as a constituent of the *Sema3b* holoreceptor. However, *Robo1* does co-immunoprecipitate with *Nrp1, Nrp2, Plxna1*, and *Plxna4*, despite only being able to bind *Nrp1* directly (Hernández-Miranda et al., 2011), This indicates that *Robo1* is at least capable of forming a complex with a *Nrp1/Nrp2* heterodimers, leaving open the possibility that *Sema3b* signals through a multimeric receptor with a composition that is as complex as the *Sema3a* receptor.

## Sema3c

Similar to *Sema3b*, *Sema3c* binds to both *Nrp1* and *Nrp2* ectopically expressed in COS and HEK-293 cells *in vitro* (Chen et al., 1997; Feiner et al., 1997; Takahashi et al., 1998; Rohm et al., 2000). Expression of a dominant negative *Nrp1* receptor (lacking extracellular domain) in embryonic chick sympathetic neurons abrogates *Sema3c* induced growth cone collapse (Renzi et al., 1999), and *Sema3c* acts as a competitive antagonist to *Sema3a* in chick embryonic retinal ganglion cells that express *Nrp1* but not *Npr2* (Takahashi et al., 1998). Also, COS cells ectopically expressing *Plxna1*/*Plxna2*/*Plxna3* and either *Nrp1* or *Nrp2* do not collapse in the presence of exogenous *Sema3c*, but do collapse in the presence of either *Sema3a* or *Sema3f* (Takahashi et al., 1999; Takahashi and Strittmatter, 2001). Furthermore when embryonic chick DRG, which normally express *Nrp1* but not *Nrp2*, are transduced to ectopically express *Nrp2* they become responsive to *Sema3c* induced growth cone collapse (Takahashi et al., 1998). Thus the holoreceptor for *Sema3c* requires both *Nrp1* and *Nrp2* to be present.

There is little evidence that *Plxna1* is essential for *Sema3c* signal transduction. Unlike for *Sema3a* and *Sema3f*, *Plxna1* co-expression in COS cells with either *Nrp1* or *Nrp2* does not increase the binding affinity for *Sema3c*, and there is no collapse response (Takahashi et al., 1999; Rohm et al., 2000; Gitler et al., 2004). Conversely, there is some evidence that *Plxna2* is important for *Sema3c* signaling; *Plxna2* positive mouse cardiac neural crest cells show aberrant migration in *Sema3c* knockouts (Brown et al., 2001). However, the same line of evidence suggests that *Nrp2* is not important for the migration of mouse cardiac neural crest cells (Chen et al., 1997), even though it has been shown that *Nrp2* is essential for *Sema3c* signaling, at least in neurons (see above). Furthermore, *Plxna2* co-expression with *Nrp2* in COS cells does not increase the affinity of *Sema3c* binding above expression of *Nrp2* alone (Rohm et al., 2000). It must be noted that binding analyses conducted so far have studied *Plxna1* and *Plxna2* in the presence of either *Nrp1* or *Nrp2*, but not *Nrp1* and *Nrp2* together. It is possible that *Plxna1* and *Plxna2* can change the binding affinity of *Sema3c* to its receptor when both *Nrp1* and *Nrp2* are present, especially as both are essential for functional transduction of the *Sema3c* signal.

Unlike *Plxna1* and *Plxna2*, the interaction of *Plxnd1* with either *Nrp1* or *Nrp2* does increase their binding affinity *Sema3c* (Gitler et al., 2004). However, *Plxnd1* knockouts show defects in the cardiac outflow tract that are remarkably similar to those seen in *Sema3c* knockouts (Gitler et al., 2004). While, unlike *Sema3e*, *Sema3c* cannot bind directly to *Plxnd1 in vitro* (Gu et al., 2005), both *Nrp1* and *Nrp2* bind to *Plxnd1* as demonstrated by co-immunoprecipitation (Gitler et al., 2004; Chauvet et al., 2007), and *Plxnd1* may form a part of the *Sema3c* receptor complex via this interaction.

As with *Sema3b*, there is no direct evidence that *Robo1* is a constituent of the *Sema3b* holoreceptor. However, because *Robo1* co-immunoprecipitates with receptor components of the *Sema3c* receptor it is possible that *Robo1* forms a complex within the *Sema3c* holoreceptor (Hernández-Miranda et al., 2011).

## Sema3d

Little is known about the receptor for *Sema3d*. What is known is that *Sema3d* can bind to *Nrp1* ectopically expressed by COS cells *in vitro* (Feiner et al., 1997). There is also some evidence *in vivo* that *Nrp1* is important for *Sema3d* signaling in zebrafish, as *Nrp1* knockdowns phenocopy *Sema3d* knockdowns; losing axon repulsion of axons from the nucleus of the medial longitudinal fasciculus (Wolman et al., 2004). The same study suggested that *Nrp2* may be a constituent of the *Sema3d* receptor, as knock-down of *Sema3d* or *Nrp2* showed a similar phenotype. It was also initially thought that *Sema3d* influenced fasciculation of axons in the nucleus of the medial longitudinal fasciculus through a receptor incorporating *Nrp1*, but it was later revealed that fasciculation is influenced by *Sema3d* modulating expression of *L1cam* on the axons (Wolman et al., 2007).

## Sema3e

Unlike all other Sema3s, *Sema3e* can bind to a Plexin, *Plxnd1*, directly and independently of the Neuropilins. Also, exogenous *Sema3e* collapses COS cells expressing ectopic *Plxnd1* (Gu et al., 2005). Further *in vitro* evidence implicating *Plxnd1* as an essential component of the *Sema3e* receptor is that *Sema3e* mediated axon growth inhibition and growth cone collapse of embryonic mouse cortical neurons is abolished in both *Plxnd1* knockout and knockdown models (Chauvet et al., 2007). Furthermore, *Plxnd1* expression is essential for *Sema3e* mediated modelling of chick vasculature both *in vivo*, and *in vitro* (Gu et al., 2005), and observed metastatic activity of *Sema3e* is dependent on activation of *Plxnd1* associated *ErbB2*/*Neu* oncogenic kinase (Casazza et al., 2010).

While *Sema3e* is able to exert a biological effect independent of Neuropilins, there is evidence that *Nrp1* is able to modulate *Sema3e* signaling, because *Sema3e* induced growth cone collapse of embryonic mouse DRG is inhibited by the addition of anti-*Nrp1* antibody (Miyazaki et al., 1999). Similarly, *Sema3e* induced neurite growth from subicular neurons *in vitro* is abolished by treatment with anti-*Nrp1* functional blocking antibody, and by knockdown of *Nrp1* (Chauvet et al., 2007). Interestingly, after *Nrp1* gain of function, cortical neurons convert their response to *Sema3e in vitro*, from neurite inhibition to neurite extension, and this sensitivity is still able to be abolished by knockdown of *Plxnd1* (Chauvet et al., 2007). The presence of *Nrp1* in the *Sema3e* receptor complex may “gate” cellular response between attractive and repulsive, through interaction of the extracellular domains of *Plxnd1* and *Nrp2* (Chauvet et al., 2007).

## Sema3f

*Sema3f* is one of the most studied of the Sema3s and its receptor complex is one of the best understood.

### Neuropilins

*Sema3f* binds to COS cells ectopically expressing *Nrp1*, with a similar affinity as *Sema3a* binding to *Nrp1* (Chen et al., 1997). *In vitro*, exogenous *Sema3f* inhibits cell attachment and spreading in the breast cancer cell line MCF7, a line that expresses *Nrp1* but not *Nrp2*; this inhibition is blocked by the addition of anti-*Nrp1* functional blocking antibody (Nasarre et al., 2003). However, exogenous *Sema3f* is unable to cause contraction of COS cells *in vitro* expressing *Nrp1* or *Nrp1* and one of *Plxna1*/*Plxna2*/*Plxna3* (Takahashi and Strittmatter, 2001), raising the possibility that *Sema3f* signaling through *Nrp1* requires the presence of an as yet unidentified co-receptor.

Intriguingly C100, a breast cancer cell line that expresses both *Nrp1* and *Nrp2*, responds to exogenous *Sema3f* with inhibition of cell spreading, and this response is insensitive to the addition of anti-*Nrp1* functional blocking antibody (Nasarre et al., 2003). Similarly in embryonic rat DRG, which also express both *Nrp1* and *Nrp2*, *Sema3f* induced axon repulsion is unaffected by addition of anti-*Nrp1* functional blocking antibody, even though the same antibody abolishes the repulsive effects of *Sema3c* and *Sema3a* (Chen et al., 1998). Perhaps, at least in some cell types, when both *Nrp1* and *Nrp2* are present, the *Sema3f* receptor is preferentially composed of *Nrp2* over *Nrp1*. Indeed, the affinity for *Sema3f* for *Nrp2* is around 10 fold greater than for *Nrp1* (Chen et al., 1997).

It follows then that *Nrp1* may not be an essential component of the *Sema3f* receptor complex, as long as *Nrp2* is also present. This hypothesis is supported by the observation that embryonic chick sympathetic neurons, which normally express both *Nrp1* and *Nrp2*, undergo growth cone collapse in the presence of exogenous *Sema3f*, and over expression of a dominant-negative *Nrp1* receptor in these neurons does not remove their *Sema3f* mediated growth cone collapse, despite the same model abrogating *Sema3a* mediated growth cone collapse (Renzi et al., 1999). The obvious inference is that *Nrp1* is an essential component of the *Sema3a* but not the *Sema3f* holoreceptor.

*Sema3f* binds to COS cells that ectopically express *Nrp2* (Chen et al., 1997), and exogenous *Sema3f* induces collapse of COS cells when they express both *Nrp2* and *Plxna1* (Takahashi et al., 1999). Dopaminergic axons grown *in vitro* from the ventral tegmental area of mice are either repelled by, or attracted to, *Sema3f* depending on age, an effect which is dependent on *Nrp2* (Kolk et al., 2009). Anti-*Nrp2* functional blocking antibody also abolishes *Sema3f* induced growth cone collapse of embryonic rat sympathetic neurons, and axon repulsion in neonatal mouse cortical neurons (Chen et al., 1997; Falk et al., 2005).

Knockout models reinforce the importance of *Nrp2* in *Sema3f* signaling. Embryonic mouse neural crest cells avoid focal *Sema3f in vitro*, but this effect is mollified in neural crest cells from *Nrp2* knockout mice (Gammill et al., 2006). *In vivo*, *Sema3f* knockout models show aberrant growth in multiple *Nrp2* expressing tracts in the mouse brain (Sahay et al., 2003). Similarly, *Nrp2* and *Sema3f* knockouts demonstrate that migration of neural crest cells during mouse development is dependent on *Sema3f* signaling through *Nrp2* (Gammill et al., 2006). Knockout models also demonstrate that *Sema3f* is important for the development of dopaminergic neurons in the mouse meso-diencephalon, and that this development is dependent on *Nrp2* signaling (Kolk et al., 2009). In the olfactory bulb, *Sema3f* signaling in olfactory sensory neurons is dependent on their expression of *Nrp2* (Takeuchi et al., 2010), and *Sema3f* and *Nrp2* knockouts phenocopy each other (Cloutier et al., 2002, 2004).

### Class a plexins

There is also evidence supporting the role of *Plxna1* in the *Sema3f* receptor; COS cells expressing both *Nrp2* and *Plxna1* bind *Sema3f* with greater affinity than those expressing *Nrp2* alone (*Plxna1* alone is unable to bind *Sema3f*) (Takahashi et al., 1999). Furthermore *Nrp2* alone is insufficient to signal COS cell collapse in response to *Sema3f*, but *Nrp2* and *Plxna1* expression combined is able to transduce this signal (Takahashi et al., 1999; Takahashi and Strittmatter, 2001). Similarly, COS cells transfected with both *Nrp2* and *Plxna2*, but not *Nrp2* alone, collapse in the presence of exogenous *Sema3f* (Takahashi and Strittmatter, 2001). Consequently, at least *in vitro*, *Plxna1* and *Plxna2* are parts of a functioning *Sema3f* receptor complex.

*Plxna3* also appears to be a part of the *Sema3f* holoreceptor. When *Plxna3* is knocked out, mouse sympathetic and hippocampal neurons lose sensitivity to the repulsive effect of *Sema3f in vitro* (Cheng et al., 2001; Yaron et al., 2005; Waimey et al., 2008). Knockouts of *Plxna3* and *Sema3f* are reported to phenocopy the defects of each other in the axon guidance of facial branchiomotor neurons (Schwarz et al., 2008), and in the olfactory bulb mosaic knockouts of *Plxna3* disrupt *Nrp2* and *Sema3f* dependent olfactory sensory neuron innervation (Takeuchi et al., 2010). In culture, CA1 pyramidal neurons respond to exogenous *Sema3f* with axon branch retraction, but this effect is absent in the same neurons from *Plxna3* knockout mice (Bagri et al., 2003). However, *Nrp2* and *Plxna3* co-expression in COS cells is insufficient to generate a *Sema3f* mediated cell contraction response (Takahashi and Strittmatter, 2001), indicating that *Plxna3* requires other co-receptors to form a functional *Sema3f* receptor.

Similar to the *Sema3a* receptor, there is evidence that both *Plxna3* and *Plxna4* are partially redundant constituents in the *Sema3f* holoreceptor. Embryonic mouse sympathetic neurons lose their migratory responsiveness to a gradient of *Sema3f in vitro* when both *Plxna3* and *Plxna4* are knocked out, but not if only one or the other is absent (Waimey et al., 2008). However, it appears that *Plxna3* and *Plxna4* do not coincide within the same *Sema3f* receptor complex, because co-immunoprecipitation studies demonstrate that they do not associate with one another, and this is unaffected by the presence of either *Nrp1* or *Nrp2* (Waimey et al., 2008). Thus *Plxna3* and *Plxna4* appear redundant for each other in *Sema3f* signaling, but as separate receptor complexes, and the preferential receptor complex is one that contains *Plxna3* (Yaron et al., 2005; Schwarz et al., 2008). Indeed, some neurons may not form the *Plxna4* constituting *Sema3f* receptor. For example mouse sympathetic neurons express both *Plxna3* and *Plxna4*, however their *Sema3f* mediated growth cone collapse response is only abrogated by *Plxna3*, but not *Plxna4*, knockout (Cheng et al., 2001; Suto et al., 2005; Yaron et al., 2005).

### Nrcam

Finally, there is also evidence that *Nrcam* mediates *Sema3f* signaling. Mouse piriform cortical neuron growth cones collapse in the presence of *Sema3f in vitro* but this effect is abolished in the presence of soluble *Nrcam*-Fc or anti-*Nrcam* antibody (Falk et al., 2005). *Nrcam* associates with *Nrp2*, and when *Nrcam* is knocked out, thalamic neurons lose their sensitivity to *Sema3f* (Demyanenko et al., 2011).

## Sema3g

*Sema3g* is the most recently discovered member of the Sema3s (Stevens and Halloran, 2005; Taniguchi et al., 2005). While there is growing evidence for the importance of *Sema3g* in cancer biology, cell migration, and axon guidance (Taniguchi et al., 2005; Bron et al., 2007; Karayan-Tapon et al., 2008; Kigel et al., 2008; Neufeld and Kessler, 2008), relatively little is known about its signaling mechanisms.

Taniguchi et al. (2005) reported that, *Sema3g* binds to COS cells expressing *Nrp2*, but not those expressing *Nrp1*. In the same study the researchers found that exogenous *Sema3g* repelled sympathetic axons which express *Nrp2*, but had no attractive/repulsive effect on dorsal root ganglion axons that don't express *Nrp2*. It can be inferred from these initial studies that *Sema3g* acts through *Nrp2*, but not *Nrp1*.

## Discussion

Our current understanding of the receptors for the Sema3s has come a long way since the first discovery of *Nrp1* and *Nrp2*. However, there is still a great deal that remains unclear. It is evident from the data reviewed above that there is not a single holoreceptor for each Sema3, and the make-up of a particular receptor complex depends on cell type, and perhaps also on the phenotypic status of the cell.

The methods used in the studies detailed in this review remain useful for further research into the specific receptor subunits that make up the receptor complexes for each Sema3. However, it is also important to understand the physical basis of the interactions between the Sema3 ligands, receptors, and co-receptors. This could contribute to our understanding of why different combinations of receptor components are necessary for binding and signaling of each Sema3. An example of how our knowledge of the physical interaction of different receptor subunits has led to a better understanding of how a Sema3 receptor complex functions is available from studies of the *Sema3a*/*Nrp1*/*Plxna1* complex.

Takahashi and Strittmatter (2001) put forward a model where *Plxna1* is constitutively inhibited by its Sema domain, and binding of both *Nrp1* and *Sema3a* together causes a conformational change, removing the inhibition, and allowing downstream signaling. Also, within the Sema domain of the Sema3s, there is a 70 amino acid stretch that is responsible for the specificity in the Sema3s (Koppel et al., 1997). From this, and Takahashi and Strittmatter's model, it is probable that the specificity within that region involves both the ability of each Sema3 to bind specific Neuropilins, and co-receptors such as *Plxna1* (Antipenko et al., 2003; Love et al., 2003; Liu et al., 2010). Interestingly, there is a conserved residue (K108) among all vertebrate Semaphorins that abuts the aforementioned 70 amino acid region, and when mutated in *Sema3a* and *Sema3f*, abrogates signaling without affecting binding to *Nrp1* and *Nrp2*, respectively, (Merte et al., 2010). The same mutation in *Sema3e* does not affect that molecule's binding or signaling through *Plxnd1*, indicating that if this mutation is having an effect on Semaphorin-Plexin binding, it is only apparent in Semaphorin-Neuropilin-Plexin complexes. Indeed, the K108N *Sema3a* mutation did not affect binding to *Nrp1*, but did reduce binding to embryonic mouse DRG growth cones, indicating that even though the residue is strongly conserved, its effect is only observed in proximity to a Neuropilin. The above evidence supports Takahashi and Strittmatter's hypothesis, and also indicates that specific regions of the Sema3 ligands affect recruitment and/or activation of their co-receptors. Indeed, it appears that that Sema3 binding to Neuropilins, and Sema3 interaction with Plexins are on quite separate regions of the Sema3 protein, and targeting of these regions may allow specific interference with Sema3 signaling.

There are also several other issues relating to Sema3 receptors that are outside the purpose of this review, but merit comment. Firstly several of the receptor components of the Sema3 receptor also act as receptors for other classes of Semaphorins (Toyofuku et al., 2004; Suto et al., 2005; Yoshida et al., 2006; Suto et al., 2007; Matsuoka et al., 2011a,b; Taniguchi et al., 2011), and even other families of proteins such as the vascular endothelial growth factors (VEGFs) (Soker et al., 1998; Neufeld et al., 2002; Guttmann-Raviv et al., 2007; Geretti et al., 2008; Zachary et al., 2009), hepatocyte growth factor (HGF) (Sulpice et al., 2008; Zachary et al., 2009), and transforming growth factor β1 (TGFβ1) (Glinka and Prud'homme, 2008). Secondly, it remains to be determined how endogenous soluble forms of the Neuropilins and *L1cam* interact with Sema3 signaling (Gagnon et al., 2000; Rossignol et al., 2000; Castellani et al., 2002; Lu et al., 2009). Thirdly, the Sema3s interact with chondroitin sulphate proteoglycans (CSPGs) in the extracellular matrix (ECM), and this interaction appears to potentiate its repulsive activity (De Wit et al., 2005; Zimmer et al., 2010). Lastly, it is unclear how a particular cell or tissue regulates its expression of the different components of the Sema3 receptor complex.

If components of the Sema3 receptor, especially the binding subunits the Neuropilins, act as receptors for other molecules, does that mean the Sema3s can act as antagonists against those other molecules or *vice versa*? It is known that Sema3 signaling involves endocytosis of the receptor complex (Castellani et al., 2004; Tojima et al., 2010), as does VEGF signaling through *Nrp1*, albeit by a different mechanism (Narazaki and Tosato, 2006; Salikhova et al., 2008). Thus it is possible that by sequestering essential receptor subunits, both VEGFs and Sema3s can act as antagonists (Narazaki and Tosato, 2006; Narazaki et al., 2008). Indeed, antagonism between Sema3s and VEGFs has been observed (Nasarre et al., 2003; Geretti et al., 2008). Furthermore, competitive inhibition of Sema3s on VEGF binding of *Nrp2* has been reported (Nasarre et al., 2005; Geretti et al., 2007), whereas it appears that Sema3s and VEGFs do not directly compete for binding to *Nrp1* (Appleton et al., 2007). In other words, we should view the Sema3s not only as ligands for their receptors, but also as possible antagonists of molecules for which they share receptor components (Takahashi et al., 1998; Parker et al., 2010).

Soluble truncated Neuropilins and *L1cam* have been identified, and observed *in vivo* (Mechtersheimer et al., 2001; Lu et al., 2009). Soluble Neuropilins can bind *VEGF*_165_ (Gagnon et al., 2000), so it is possible then that they are also able to bind and sequester Sema3s from functional receptors. These soluble Neuropilins do not form part of the Sema3s's holoreceptors, but they are receptors for the Sema3s. Future studies into the effect of the Sema3s should be mindful of the presence of these soluble Neuropilins, and their buffering effect taken into account when considering ligand/receptor binding. Similar note should be made of *L1cam* expression, as its soluble form is known to modulate at least *Sema3a* signaling (Castellani et al., 2000, 2002, 2004). Another intriguing finding is that soluble amyloid precursor protein can bind *Sema3a* (Magdesian et al., 2011), and may act similar to soluble Neuropilins as an inhibitor to Sema3 signaling.

Based on the interaction of *Sema3a* with CSPGs (De Wit et al., 2005; Zimmer et al., 2010), Zimmer et al. (2010) speculate that amongst other possible mechanisms, CSPGs may interact directly with CAMs in the *Sema3a* receptor complex. CSPGs in the ECM could also act to stabilize *Sema3a* for presentation to *Sema3a* sensitive cells (De Wit et al., 2005). Interestingly, the presence of heparin and heparan sulphates, but not chondroitin sulphates, enhances binding and activity of *Sema3a* to *Nrp1* expressing cells (De Wit et al., 2005), which may be related to the heparin binding site on *Nrp1* (Vander Kooi et al., 2007). Also, via an as yet unexplained mechanism, *Sema3c* release from proteoglycans in the ECM *in vitro* increases its cell migration effect on MCF7 cells, despite no change in observed binding of *Sema3c* to the cell surface (Esselens et al., 2010). These studies show that in some respects, proteoglycans in the ECM can act as “helper” receptors for Sema3s, and may play an important role in Sema3 signaling *in vivo*.

Finally, factors that influence the regulation of expression of the Sema3 receptor components are still far from understood. It is known, for example, that there are changes in expression of the Neuropilins and PlexinAs during development, after injury, and in response to at least some growth factors (de Winter et al., 2002, 2004; Banerjee et al., 2006). Further factors that regulate expression of both Neuropilins are summarised by Bielenberg et al. (2006). VEGF regulates the expression of *Plxnd1* in the endothelial cells in the developing mouse retina (Kim et al., 2011). In *Xenopus laevis*, fibroblast growth factor regulates expression of *Sema3a* (Atkinson-Leadbeater et al., 2010). It is also known that *Sema3d* can modulate expression of *L1cam*, (Wolman et al., 2004), and that *Robo1* similarly affects expression of *Nrp1* and *Plxna1* (Hernández-Miranda et al., 2011). Interestingly, *Nrcam* sensitises commissural axons to *Sema3b* by inhibiting the proteolytic degradation of another receptor component, *Plxna1* (Nawabi et al., 2010). Furthermore, *Sema3a* can induce protein synthesis at the growth cone via upregulation of translation (Campbell and Holt, 2001), and this could modulate expression of a wide range of proteins, including receptors. A deeper understanding of how the expression of Sema3 receptors is regulated will provide insight into the molecular mechanisms that cause aberrant expression or receptor components in pathological situations.

This review brings together the growing number of investigations into what receptor subunits are important for each Sema3 ligand. It is apparent that there is still much to learn about the Sema3 receptors, and that it is important we gain a fuller understanding of the Sema3 receptor complexes. When combined with greater knowledge of the signaling cascades involved with each receptor subunit, there is the tantalising possibility of designing therapies for the increasing number of pathological situations in which Sema3s and their receptors are implicated.

### Conflict of interest statement

The authors declare that the research was conducted in the absence of any commercial or financial relationships that could be construed as a potential conflict of interest.

## References

[B1] Antipenko A. Himanen J. P. Van Leyen K. Nardi-Dei V. Lesniak J. Barton W. A. Rajashankar K. R. Lu M. Hoemme C. Püschel A. W. Nikolov D. B. (2003). Structure of the semaphorin-3A receptor binding module. Neuron 39, 589–598 10.1016/S0896-6273(03)00502-6 12925274

[B2] Appleton B. A. Wu P. Maloney J. Yin J. Liang W. C. Stawicki S. Mortara K. Bowman K. K. Elliott J. M. Desmarais W. Bazan J. F. Bagri A. Tessier-Lavigne M. Koch A. W. Wu Y. Watts R. J. Wiesmann C. (2007). Structural studies of neuropilin/antibody complexes provide insights into semaphorin and VEGF binding. EMBO J. 26, 4902–4912 10.1038/sj.emboj.7601906 17989695 PMC2099469

[B3] Atkinson-Leadbeater K. Bertolesi G. E. Hehr C. L. Webber C. A. Cechmanek P. B. McFarlane S. (2010). Dynamic expression of axon guidance cues required for optic tract development is controlled by fibroblast growth factor signaling. J. Neurosci. 30, 685–693 10.1523/JNEUROSCI.4165-09.2010 20071533 PMC6633001

[B4] Bagri A. Cheng H. J. Yaron A. Pleasure S. J. Tessier-Lavigne M. (2003). Stereotyped pruning of long hippocampal axon branches triggered by retraction inducers of the semaphorin family. Cell 113, 285–299 10.1016/S0092-8674(03)00267-8 12732138

[B5] Banerjee S. Sengupta K. Dhar K. Mehta S. D'Amore P. A. Dhar G. Banerjee S. K. (2006). Breast cancer cells secreted platelet-derived growth factor-induced motility of vascular smooth muscle cells is mediated through neuropilin-1. Mol. Carcinog. 45, 871–880 10.1002/mc.20248 16847823

[B6] Bechara A. Nawabi H. Moret F. Yaron A. Weaver E. Bozon M. Abouzid K. Guan J. L. Tessier-Lavigne M. Lemmon V. Castellani V. (2008). FAK-MAPK-dependent adhesion disassembly downstream of L1 contributes to semaphorin3A-induced collapse. EMBO J. 27, 1549–1562 10.1038/emboj.2008.86 18464795 PMC2426724

[B7] Ben-Zvi A. Manor O. Schachner M. Yaron A. Tessier-Lavigne M. Behar O. (2008). The Semaphorin receptor PlexinA3 mediates neuronal apoptosis during dorsal root ganglia development. J. Neurosci. 28, 12427–12432 10.1523/JNEUROSCI.3573-08.2008 19020035 PMC6671732

[B8] Bielenberg D. R. Pettaway C. A. Takashima S. Klagsbrun M. (2006). Neuropilins in neoplasms: expression, regulation, and function. Exp. Cell Res. 312, 584–593 10.1016/j.yexcr.2005.11.024 16445911

[B9] Bron R. Vermeren M. Kokot N. Andrews W. Little G. E. Mitchell K. J. Cohen J. (2007). Boundary cap cells constrain spinal motor neuron somal migration at motor exit points by a semaphorin-plexin mechanism. Neural Dev. 2, 21 10.1186/1749-8104-2-21 17971221 PMC2131750

[B10] Brown C. B. Feiner L. Lu M. M. Li J. Ma X. Webber A. L. Jia L. Raper J. A. Epstein J. A. (2001). PlexinA2 and semaphorin signaling during cardiac neural crest development. Development 128, 3071–3080 11688557 10.1242/dev.128.16.3071

[B11] Campbell D. S. Holt C. E. (2001). Chemotropic responses of retinal growth cones mediated by rapid local protein synthesis and degradation. Neuron 32, 1013–1026 10.1016/S0896-6273(01)00551-7 11754834

[B12] Campbell D. S. Regan A. G. Lopez J. S. Tannahill D. Harris W. A. Holt C. E. (2001). Semaphorin 3A elicits stage-dependent collapse, turning, and branching in Xenopus retinal growth cones. J. Neurosci. 21, 8538–8547 11606642 10.1523/JNEUROSCI.21-21-08538.2001 PMC6762807

[B13] Cariboni A. Davidson K. Rakic S. Maggi R. Parnavelas J. G. Ruhrberg C. (2011). Defective gonadotropin-releasing hormone neuron migration in mice lacking SEMA3A signalling through NRP1 and NRP2: implications for the aetiology of hypogonadotropic hypogonadism. Hum. Mol. Genet. 20, 336–344 10.1093/hmg/ddq468 21059704

[B14] Casazza A. Finisguerra V. Capparuccia L. Camperi A. Swiercz J. M. Rizzolio S. Rolny C. Christensen C. Bertotti A. Sarotto I. Risio M. Trusolino L. Weitz J. Schneider M. Mazzone M. Comoglio P. M. Tamagnone L. (2010). Sema3E-Plexin D1 signaling drives human cancer cell invasiveness and metastatic spreading in mice. J. Clin. Invest. 120, 2684–2698 10.1172/JCI42118 20664171 PMC2912191

[B15] Castellani V. Chedotal A. Schachner M. Faivre-Sarrailh C. Rougon G. (2000). Analysis of the L1-deficient mouse phenotype reveals cross-talk between Sema3A and L1 signaling pathways in axonal guidance. Neuron 27, 237–249 10.1016/S0896-6273(00)00033-7 10985345

[B16] Castellani V. De Angelis E. Kenwrick S. Rougon G. (2002). Cis and trans interactions of L1 with neuropilin-1 control axonal responses to semaphorin 3A. EMBO J. 21, 6348–6357 12456642 10.1093/emboj/cdf645 PMC136949

[B17] Castellani V. Falk J. Rougon G. (2004). Semaphorin3A-induced receptor endocytosis during axon guidance responses is mediated by L1 CAM. Mol. Cell. Neurosci. 26, 89–100 10.1016/j.mcn.2004.01.010 15121181

[B18] Chauvet S. Cohen S. Yoshida Y. Fekrane L. Livet J. Gayet O. Segu L. Buhot M. C. Jessell T. M. Henderson C. E. Mann F. (2007). Gating of Sema3E/PlexinD1 signaling by neuropilin-1 switches axonal repulsion to attraction during brain development. Neuron 56, 807–822 10.1016/j.neuron.2007.10.019 18054858 PMC2700040

[B19] Chen H. Chedotal A. He Z. Goodman C. S. Tessier-Lavigne M. (1997). Neuropilin-2, a novel member of the neuropilin family, is a high affinity receptor for the semaphorins Sema E and Sema IV but not Sema III. Neuron 19, 547–559 10.1016/S0896-6273(00)80371-2 9331348

[B20] Chen H. He Z. Bagri A. Tessier-Lavigne M. (1998). Semaphorin-neuropilin interactions underlying sympathetic axon responses to class III semaphorins. Neuron 21, 1283–1290 10.1016/S0896-6273(00)80648-0 9883722

[B21] Cheng H. J. Bagri A. Yaron A. Stein E. Pleasure S. J. Tessier-Lavigne M. (2001). Plexin-A3 mediates semaphorin signaling and regulates the development of hippocampal axonal projections. Neuron 32, 249–263 10.1016/S0896-6273(01)00478-0 11683995

[B22] Cloutier J. F. Giger R. J. Koentges G. Dulac C. Kolodkin A. L. Ginty D. D. (2002). Neuropilin-2 mediates axonal fasciculation, zonal segregation, but not axonal convergence, of primary accessory olfactory neurons. Neuron 33, 877–892 10.1016/S0896-6273(02)00635-9 11906695

[B23] Cloutier J. F. Sahay A. Chang E. C. Tessier-Lavigne M. Dulac C. Kolodkin A. L. Ginty D. D. (2004). Differential requirements for semaphorin 3F and Slit-1 in axonal targeting, fasciculation, and segregation of olfactory sensory neuron projections. J. Neurosci. 24, 9087–9096 10.1523/JNEUROSCI.2786-04.2004 15483127 PMC6730055

[B24] de Winter F. Cui Q. Symons N. Verhaagen J. Harvey A. R. (2004). Expression of class-3 semaphorins and their receptors in the neonatal and adult rat retina. Invest. Ophthalmol. Vis. Sci. 45, 4554–4562 10.1167/iovs.04-0173 15557467

[B25] de Winter F. Oudega M. Lankhorst A. J. Hamers F. P. Blits B. Ruitenberg M. J. Pasterkamp R. J. Gispen W. H. Verhaagen J. (2002). Injury-induced class 3 semaphorin expression in the rat spinal cord. Exp. Neurol. 175, 61–75 10.1006/exnr.2002.7884 12009760

[B26] De Wit J. De Winter F. Klooster J. Verhaagen J. (2005). Semaphorin 3A displays a punctate distribution on the surface of neuronal cells and interacts with proteoglycans in the extracellular matrix. Mol. Cell. Neurosci. 29, 40–55 10.1016/j.mcn.2004.12.009 15866045

[B27] Demyanenko G. P. Riday T. T. Tran T. S. Dalal J. Darnell E. P. Brennaman L. H. Sakurai T. Grumet M. Philpot B. D. Maness P. F. (2011). NrCAM deletion causes topographic mistargeting of thalamocortical axons to the visual cortex and disrupts visual acuity. J. Neurosci. 31, 1545–1558 10.1523/JNEUROSCI.4467-10.2011 21273439 PMC3037548

[B28] Esselens C. Malapeira J. Colome N. Casal C. Rodriguez-Manzaneque J. C. Canals F. Arribas J. (2010). The cleavage of semaphorin 3C induced by ADAMTS1 promotes cell migration. J. Biol. Chem. 285, 2463–2473 10.1074/jbc.M109.055129 19915008 PMC2807303

[B29] Falk J. Bechara A. Fiore R. Nawabi H. Zhou H. Hoyo-Becerra C. Bozon M. Rougon G. Grumet M. Püschel A. W. Sanes J. R. Castellani V. (2005). Dual functional activity of semaphorin 3B is required for positioning the anterior commissure. Neuron 48, 63–75 10.1016/j.neuron.2005.08.033 16202709

[B30] Feiner L. Koppel A. M. Kobayashi H. Raper J. A. (1997). Secreted chick semaphorins bind recombinant neuropilin with similar affinities but bind different subsets of neurons *in situ*. Neuron 19, 539–545 10.1016/S0896-6273(00)80370-0 9331347

[B31] Gagnon M. L. Bielenberg D. R. Gechtman Z. Miao H. Q. Takashima S. Soker S. Klagsbrun M. (2000). Identification of a natural soluble neuropilin-1 that binds vascular endothelial growth factor: *in vivo* expression and antitumor activity. Proc. Natl. Acad. Sci. U.S.A. 97, 2573–2578 10.1073/pnas.040337597 10688880 PMC15970

[B32] Gammill L. S. Gonzalez C. Gu C. Bronner-Fraser M. (2006). Guidance of trunk neural crest migration requires neuropilin 2/semaphorin 3F signaling. Development 133, 99–106 10.1242/dev.02187 16319111

[B33] Geretti E. Shimizu A. Klagsbrun M. (2008). Neuropilin structure governs VEGF and semaphorin binding and regulates angiogenesis. Angiogenesis 11, 31–39 10.1007/s10456-008-9097-1 18283547

[B34] Geretti E. Shimizu A. Kurschat P. Klagsbrun M. (2007). Site-directed mutagenesis in the B-neuropilin-2 domain selectively enhances its affinity to VEGF165, but not to semaphorin 3F. J. Biol. Chem. 282, 25698–25707 10.1074/jbc.M702942200 17595163

[B35] Gitler A. D. Lu M. M. Epstein J. A. (2004). PlexinD1 and semaphorin signaling are required in endothelial cells for cardiovascular development. Dev. Cell 7, 107–116 10.1016/j.devcel.2004.06.002 15239958

[B36] Glinka Y. Prud'homme G. J. (2008). Neuropilin-1 is a receptor for transforming growth factor beta-1, activates its latent form, and promotes regulatory T cell activity. J. Leukoc. Biol. 84, 302–310 10.1189/jlb.0208090 18436584 PMC2504713

[B37] Gu C. Rodriguez E. R. Reimert D. V. Shu T. Fritzsch B. Richards L. J. Kolodkin A. L. Ginty D. D. (2003). Neuropilin-1 conveys semaphorin and VEGF signaling during neural and cardiovascular development. Dev. Cell 5, 45–57 10.1016/S1534-5807(03)00169-2 12852851 PMC3918747

[B38] Gu C. Yoshida Y. Livet J. Reimert D. V. Mann F. Merte J. Henderson C. E. Jessell T. M. Kolodkin A. L. Ginty D. D. (2005). Semaphorin 3E and plexin-D1 control vascular pattern independently of neuropilins. Science 307, 265–268 10.1126/science.1105416 15550623

[B39] Guttmann-Raviv N. Shraga-Heled N. Varshavsky A. Guimaraes-Sternberg C. Kessler O. Neufeld G. (2007). Semaphorin-3A and semaphorin-3F work together to repel endothelial cells and to inhibit their survival by induction of apoptosis. J. Biol. Chem. 282, 26294–26305 10.1074/jbc.M609711200 17569671

[B40] He Z. Tessier-Lavigne M. (1997). Neuropilin is a receptor for the axonal chemorepellent Semaphorin III. Cell 90, 739–751 10.1016/S0092-8674(00)80534-6 9288753

[B41] Hernández-Miranda L. R. Cariboni A. Faux C. Ruhrberg C. Cho J. H. Cloutier J. F. Eickholt B. J. Parnavelas J. G. Andrews W. D. (2011). Robo1 regulates semaphorin signaling to guide the migration of cortical interneurons through the ventral forebrain. J. Neurosci. 31, 6174–6187 10.1523/JNEUROSCI.5464-10.2011 21508241 PMC3088089

[B42] Kapfhammer J. P. Xu H. Raper J. A. (2007). The detection and quantification of growth cone collapsing activities. Nat. Protoc. 2, 2005–2011 10.1038/nprot.2007.295 17703212

[B43] Karayan-Tapon L. Wager M. Guilhot J. Levillain P. Marquant C. Clarhaut J. Potiron V. Roche J. (2008). Semaphorin, neuropilin and VEGF expression in glial tumours: SEMA3G, a prognostic marker? Br. J. Cancer 99, 1153–1160 10.1038/sj.bjc.6604641 18781179 PMC2567090

[B44] Kawakami A. Kitsukawa T. Takagi S. Fujisawa H. (1996). Developmentally regulated expression of a cell surface protein, neuropilin, in the mouse nervous system. J. Neurobiol. 29, 1–17 10.1002/(SICI)1097-4695(199601)29:1<1::AID-NEU1>3.0.CO;2-F 8748368

[B45] Kigel B. Rabinowicz N. Varshavsky A. Kessler O. Neufeld G. (2011). Plexin-A4 promotes tumor progression and tumor angiogenesis by enhancement of VEGF and bFGF signaling. Blood 118, 4285–4296 10.1182/blood-2011-03-341388 21832283

[B46] Kigel B. Varshavsky A. Kessler O. Neufeld G. (2008). Successful inhibition of tumor development by specific class-3 semaphorins is associated with expression of appropriate semaphorin receptors by tumor cells. PLoS ONE 3:e3287 10.1371/journal.pone.0003287 18818766 PMC2538586

[B47] Kim J. Oh W. J. Gaiano N. Yoshida Y. Gu C. (2011). Semaphorin 3E-Plexin-D1 signaling regulates VEGF function in developmental angiogenesis via a feedback mechanism. Genes Dev. 25, 1399–1411 10.1101/gad.2042011 21724832 PMC3134083

[B48] Kitsukawa T. Shimizu M. Sanbo M. Hirata T. Taniguchi M. Bekku Y. Yagi T. Fujisawa H. (1997). Neuropilin-semaphorin III/D-mediated chemorepulsive signals play a crucial role in peripheral nerve projection in mice. Neuron 19, 995–1005 10.1016/S0896-6273(00)80392-X 9390514

[B49] Kolk S. M. Gunput R. A. Tran T. S. Van Den Heuvel D. M. Prasad A. A. Hellemons A. J. Adolfs Y. Ginty D. D. Kolodkin A. L. Burbach J. P. Smidt M. P. Pasterkamp R. J. (2009). Semaphorin 3F is a bifunctional guidance cue for dopaminergic axons and controls their fasciculation, channeling, rostral growth, and intracortical targeting. J. Neurosci. 29, 12542–12557 10.1523/JNEUROSCI.2521-09.2009 19812329 PMC3097132

[B50] Kolodkin A. L. Levengood D. V. Rowe E. G. Tai Y. T. Giger R. J. Ginty D. D. (1997). Neuropilin is a semaphorin III receptor. Cell 90, 753–762 10.1016/S0092-8674(00)80535-8 9288754

[B51] Kolodkin A. L. Matthes D. J. Goodman C. S. (1993). The semaphorin genes encode a family of transmembrane and secreted growth cone guidance molecules. Cell 75, 1389 10.1016/0092-8674(93)90625-Z 8269517

[B52] Kolodkin A. L. Matthes D. J. O'Connor T. P. Patel N. H. Admon A. Bentley D. Goodman C. S. (1992). Fasciclin IV: sequence, expression, and function during growth cone guidance in the grasshopper embryo. Neuron 9, 831–845 10.1016/0896-6273(92)90237-8 1418998

[B53] Koppel A. M. Feiner L. Kobayashi H. Raper J. A. (1997). A 70 amino acid region within the semaphorin domain activates specific cellular response of semaphorin family members. Neuron 19, 531–537 10.1016/S0896-6273(00)80369-4 9331346

[B54] Liu H. Juo Z. S. Shim A. H. Focia P. J. Chen X. Garcia K. C. He X. (2010). Structural basis of semaphorin-plexin recognition and viral mimicry from Sema7A and A39R complexes with PlexinC1. Cell 142, 749–761 10.1016/j.cell.2010.07.040 20727575 PMC2936782

[B55] Love C. A. Harlos K. Mavaddat N. Davis S. J. Stuart D. I. Jones E. Y. Esnouf R. M. (2003). The ligand-binding face of the semaphorins revealed by the high-resolution crystal structure of SEMA4D. Nat. Struct. Biol. 10, 843–848 10.1038/nsb977 12958590

[B56] Lu Y. Xiang H. Liu P. Tong R. R. Watts R. J. Koch A. W. Sandoval W. N. Damico L. A. Wong W. L. Meng Y. G. (2009). Identification of circulating neuropilin-1 and dose-dependent elevation following anti-neuropilin-1 antibody administration. MAbs 1, 364–369 10.4161/mabs.1.4.8885 20068394 PMC2726604

[B57] Luo Y. Raible D. Raper J. A. (1993). Collapsin: a protein in brain that induces the collapse and paralysis of neuronal growth cones. Cell 75, 217–227 10.1016/0092-8674(93)80064-L 8402908

[B58] Luo Y. Shepherd I. Li J. Renzi M. J. Chang S. Raper J. A. (1995). A family of molecules related to collapsin in the embryonic chick nervous system. Neuron 14, 1131–1140 10.1016/0896-6273(95)90261-9 7605628

[B59] Magdesian M. H. Gralle M. Guerreiro L. H. Beltrao P. J. I. Carvalho M. Santos L. E. D. De Mello F. G. Reis R. A. M. Ferreira S. T. (2011). Secreted human amyloid precursor protein binds semaphorin 3a and prevents semaphorin-induced growth cone collapse. PLoS ONE 6:e22857 10.1371/journal.pone.0022857 21829538 PMC3146505

[B60] Maretzky T. Schulte M. Ludwig A. Rose-John S. Blobel C. Hartmann D. Altevogt P. Saftig P. Reiss K. (2005). L1 is sequentially processed by two differently activated metalloproteases and presenilin/gamma-secretase and regulates neural cell adhesion, cell migration, and neurite outgrowth. Mol. Cell. Biol. 25, 9040–9053 10.1128/MCB.25.20.9040-9053.2005 16199880 PMC1265787

[B61] Matsuoka R. L. Chivatakarn O. Badea T. C. Samuels I. S. Cahill H. Katayama K. Kumar S. R. Suto F. Chedotal A. Peachey N. S. Nathans J. Yoshida Y. Giger R. J. Kolodkin A. L. (2011a). Class 5 transmembrane semaphorins control selective Mammalian retinal lamination and function. Neuron 71, 460–473 10.1016/j.neuron.2011.06.009 21835343 PMC3164552

[B62] Matsuoka R. L. Nguyen-Ba-Charvet K. T. Parray A. Badea T. C. Chedotal A. Kolodkin A. L. (2011b). Transmembrane semaphorin signalling controls laminar stratification in the mammalian retina. Nature 470, 259–263 10.1038/nature09675 21270798 PMC3063100

[B63] Mechtersheimer S. Gutwein P. Agmon-Levin N. Stoeck A. Oleszewski M. Riedle S. Postina R. Fahrenholz F. Fogel M. Lemmon V. Altevogt P. (2001). Ectodomain shedding of L1 adhesion molecule promotes cell migration by autocrine binding to integrins. J. Cell Biol. 155, 661–673 10.1083/jcb.200101099 11706054 PMC2198870

[B64] Merte J. Wang Q. Vander Kooi C. W. Sarsfield S. Leahy D. J. Kolodkin A. L. Ginty D. D. (2010). A forward genetic screen in mice identifies Sema3A(K108N), which binds to neuropilin-1 but cannot signal. J. Neurosci. 30, 5767–5775 10.1523/JNEUROSCI.5061-09.2010 20410128 PMC2869466

[B65] Messersmith E. K. Leonardo E. D. Shatz C. J. Tessier-Lavigne M. Goodman C. S. Kolodkin A. L. (1995). Semaphorin III can function as a selective chemorepellent to pattern sensory projections in the spinal cord. Neuron 14, 949 10.1016/0896-6273(95)90333-X 7748562

[B66] Miyazaki N. Furuyama T. Sakai T. Fujioka S. Mori T. Ohoka Y. Takeda N. Kubo T. Inagaki S. (1999). Developmental localization of semaphorin H messenger RNA acting as a collapsing factor on sensory axons in the mouse brain. Neuroscience 93, 401–408 10.1016/S0306-4522(99)00134-7 10430503

[B67] Nakamura F. Tanaka M. Takahashi T. Kalb R. G. Strittmatter S. M. (1998). Neuropilin-1 extracellular domains mediate semaphorin D/III-induced growth cone collapse. Neuron 21, 1093–1100 10.1016/S0896-6273(00)80626-1 9856464

[B68] Narazaki M. Segarra M. Tosato G. (2008). Sulfated polysaccharides identified as inducers of neuropilin-1 internalization and functional inhibition of VEGF165 and semaphorin3A. Blood 111, 4126–4136 10.1182/blood-2007-09-112474 18272814 PMC2288723

[B69] Narazaki M. Tosato G. (2006). Ligand-induced internalization selects use of common receptor neuropilin-1 by VEGF165 and semaphorin3A. Blood 107, 3892–3901 10.1182/blood-2005-10-4113 16424390 PMC1895286

[B70] Nasarre C. Koncina E. Labourdette G. Cremel G. Roussel G. Aunis D. Bagnard D. (2009). Neuropilin-2 acts as a modulator of Sema3A-dependent glioma cell migration. Cell Adh. Migr. 3, 383–389 10.4161/cam.3.4.9934 19855168 PMC2802752

[B71] Nasarre P. Constantin B. Rouhaud L. Harnois T. Raymond G. Drabkin H. A. Bourmeyster N. Roche J. (2003). Semaphorin SEMA3F and VEGF have opposing effects on cell attachment and spreading. Neoplasia 5, 83–92 12659673 10.1016/s1476-5586(03)80020-9 PMC1502125

[B72] Nasarre P. Kusy S. Constantin B. Castellani V. Drabkin H. A. Bagnard D. Roche J. (2005). Semaphorin SEMA3F has a repulsing activity on breast cancer cells and inhibits E-cadherin-mediated cell adhesion. Neoplasia 7, 180–189 10.1593/neo.04481 15802023 PMC1501131

[B73] Nawabi H. Briancon-Marjollet A. Clark C. Sanyas I. Takamatsu H. Okuno T. Kumanogoh A. Bozon M. Takeshima K. Yoshida Y. Moret F. Abouzid K. Castellani V. (2010). A midline switch of receptor processing regulates commissural axon guidance in vertebrates. Genes Dev. 24, 396–410 10.1101/gad.542510 20159958 PMC2816738

[B74] Neufeld G. Cohen T. Shraga N. Lange T. Kessler O. Herzog Y. (2002). The neuropilins: multifunctional semaphorin and VEGF receptors that modulate axon guidance and angiogenesis. Trends Cardiovasc. Med. 12, 13–19 11796239 10.1016/s1050-1738(01)00140-2

[B75] Neufeld G. Kessler O. (2008). The semaphorins: versatile regulators of tumour progression and tumour angiogenesis. Nat. Rev. Cancer 8, 632–645 10.1038/nrc2404 18580951

[B76] Parker M. W. Hellman L. M. Xu P. Fried M. G. Vander Kooi C. W. (2010). Furin processing of semaphorin 3F determines its anti-angiogenic activity by regulating direct binding and competition for neuropilin. Biochemistry 49, 4068–4075 10.1021/bi100327r 20387901 PMC2868107

[B77] Pasterkamp R. J. Kolodkin A. L. (2003). Semaphorin junction: making tracks toward neural connectivity. Curr. Opin. Neurobiol. 13, 79–89 10.1016/S0959-4388(03)00003-5 12593985

[B78] Pellet-Many C. Frankel P. Jia H. Zachary I. (2008). Neuropilins: structure, function and role in disease. Biochem. J. 411, 211–226 10.1042/BJ20071639 18363553

[B79] Püschel A. W. Adams R. H. Betz H. (1995). Murine semaphorin D/collapsin is a member of a diverse gene family and creates domains inhibitory for axonal extension. Neuron 14, 941–948 10.1016/0896-6273(95)90332-1 7748561

[B80] Raper J. A. (2000). Semaphorins and their receptors in vertebrates and invertebrates. Curr. Opin. Neurobiol. 10, 88–94 10.1016/S0959-4388(99)00057-4 10679438

[B81] Renzi M. J. Feiner L. Koppel A. M. Raper J. A. (1999). A dominant negative receptor for specific secreted semaphorins is generated by deleting an extracellular domain from neuropilin-1. J. Neurosci. 19, 7870–7880 10479689 10.1523/JNEUROSCI.19-18-07870.1999 PMC6782461

[B82] Roche J. Boldog F. Robinson M. Robinson L. Varella-Garcia M. Swanton M. Waggoner B. Fishel R. Franklin W. Gemmill R. Drabkin H. (1996). Distinct 3p21.3 deletions in lung cancer and identification of a new human semaphorin. Oncogene 12, 1289–1297 8649831

[B83] Rohm B. Ottemeyer A. Lohrum M. Püschel A. W. (2000). Plexin/neuropilin complexes mediate repulsion by the axonal guidance signal semaphorin 3A. Mech. Dev. 93, 95–104 10.1016/S0925-4773(00)00269-0 10781943

[B84] Rossignol M. Gagnon M. L. Klagsbrun M. (2000). Genomic organization of human neuropilin-1 and neuropilin-2 genes: identification and distribution of splice variants and soluble isoforms. Genomics 70, 211–222 10.1006/geno.2000.6381 11112349

[B85] Roth L. Koncina E. Satkauskas S. Cremel G. Aunis D. Bagnard D. (2009). The many faces of semaphorins: from development to pathology. Cell. Mol. Life Sci. 66, 649–666 10.1007/s00018-008-8518-z 18953684 PMC11131483

[B86] Roth L. Nasarre C. Dirrig-Grosch S. Aunis D. Cremel G. Hubert P. Bagnard D. (2008). Transmembrane domain interactions control biological functions of neuropilin-1. Mol. Biol. Cell 19, 646–654 10.1091/mbc.E07-06-0625 18045991 PMC2230599

[B87] Sahay A. Molliver M. E. Ginty D. D. Kolodkin A. L. (2003). Semaphorin 3F is critical for development of limbic system circuitry and is required in neurons for selective CNS axon guidance events. J. Neurosci. 23, 6671–6680 12890759 10.1523/JNEUROSCI.23-17-06671.2003 PMC6740712

[B88] Sakurai A. Doci C. L. Gutkind J. S. (2012). Semaphorin signaling in angiogenesis, lymphangiogenesis and cancer. Cell Res. 22, 23–32 10.1038/cr.2011.198 22157652 PMC3351930

[B89] Salikhova A. Wang L. Lanahan A. A. Liu M. Simons M. Leenders W. P. Mukhopadhyay D. Horowitz A. (2008). Vascular endothelial growth factor and semaphorin induce neuropilin-1 endocytosis via separate pathways. Circ. Res. 103, e71–e79 10.1161/CIRCRESAHA.108.183327 18723443 PMC2674948

[B90] Schlatter M. C. Buhusi M. Wright A. G. Maness P. F. (2008). CHL1 promotes Sema3A-induced growth cone collapse and neurite elaboration through a motif required for recruitment of ERM proteins to the plasma membrane. J. Neurochem. 104, 731–744 10.1111/j.1471-4159.2007.05013.x 17995939

[B91] Schwarz Q. Waimey K. E. Golding M. Takamatsu H. Kumanogoh A. Fujisawa H. Cheng H. J. Ruhrberg C. (2008). Plexin A3 and plexin A4 convey semaphorin signals during facial nerve development. Dev. Biol. 324, 1–9 10.1016/j.ydbio.2008.08.020 18804103 PMC2814064

[B92] Sekido Y. Bader S. Latif F. Chen J.-Y. Duh F.-M. Wei M.-H. Albanesi J. P. Lee C.-C. Lerman M. I. Minna J. D. (1996). Human semaphorins A(V) and IV reside in the 3p21.3 small cell lung cancer deletion region and demonstrate distinct expression patterns. Proc. Natl. Acad. Sci. U.S.A. 93, 4120–4125 8633026 10.1073/pnas.93.9.4120 PMC39497

[B93] Semaphorin Nomenclature Committee. (1999). Unified nomenclature for the semaphorins/collapsins. Cell 97, 551–552 10.1016/S0092-8674(00)80766-7 10367884

[B94] Soker S. Takashima S. Miao H. Q. Neufeld G. Klagsbrun M. (1998). Neuropilin-1 is expressed by endothelial and tumor cells as an isoform-specific receptor for vascular endothelial growth factor. Cell 92, 735–745 10.1016/S0092-8674(00)81402-6 9529250

[B95] Staton C. A. (2011). Class 3 semaphorins and their receptors in physiological and pathological angiogenesis. Biochem. Soc. Trans. 39, 1565–1570 10.1042/BST20110654 22103488

[B96] Stevens C. B. Halloran M. C. (2005). Developmental expression of sema3G, a novel zebrafish semaphorin. Gene Expr. Patterns 5, 647–653 10.1016/j.modgep.2005.02.009 15939377

[B97] Sulpice E. Plouet J. Berge M. Allanic D. Tobelem G. Merkulova-Rainon T. (2008). Neuropilin-1 and neuropilin-2 act as coreceptors, potentiating proangiogenic activity. Blood 111, 2036–2045 10.1182/blood-2007-04-084269 18065694

[B98] Suto F. Ito K. Uemura M. Shimizu M. Shinkawa Y. Sanbo M. Shinoda T. Tsuboi M. Takashima S. Yagi T. Fujisawa H. (2005). Plexin-a4 mediates axon-repulsive activities of both secreted and transmembrane semaphorins and plays roles in nerve fiber guidance. J. Neurosci. 25, 3628–3637 10.1523/JNEUROSCI.4480-04.2005 15814794 PMC6725384

[B99] Suto F. Tsuboi M. Kamiya H. Mizuno H. Kiyama Y. Komai S. Shimizu M. Sanbo M. Yagi T. Hiromi Y. Chedotal A. Mitchell K. J. Manabe T. Fujisawa H. (2007). Interactions between plexin-A2, plexin-A4, and semaphorin 6A control lamina-restricted projection of hippocampal mossy fibers. Neuron 53, 535–547 10.1016/j.neuron.2007.01.028 17296555

[B100] Takahashi T. Fournier A. Nakamura F. Wang L. H. Murakami Y. Kalb R. G. Fujisawa H. Strittmatter S. M. (1999). Plexin-neuropilin-1 complexes form functional semaphorin-3A receptors. Cell 99, 59–69 10.1016/S0092-8674(00)80062-8 10520994

[B101] Takahashi T. Nakamura F. Jin Z. Kalb R. G. Strittmatter S. M. (1998). Semaphorins A and E act as antagonists of neuropilin-1 and agonists of neuropilin-2 receptors. Nat. Neurosci. 1, 487–493 10.1038/2203 10196546

[B102] Takahashi T. Strittmatter S. M. (2001). Plexina1 autoinhibition by the plexin sema domain. Neuron 29, 429–439 10.1016/S0896-6273(01)00216-1 11239433

[B103] Takamatsu H. Kumanogoh A. (2012). Diverse roles for semaphorin-plexin signaling in the immune system. Trends Immunol. 33, 127–135 10.1016/j.it.2012.01.008 22325954

[B104] Takeuchi H. Inokuchi K. Aoki M. Suto F. Tsuboi A. Matsuda I. Suzuki M. Aiba A. Serizawa S. Yoshihara Y. Fujisawa H. Sakano H. (2010). Sequential arrival and graded secretion of Sema3F by olfactory neuron axons specify map topography at the bulb. Cell 141, 1056–1067 10.1016/j.cell.2010.04.041 20550939

[B105] Takegahara N. Kumanogoh A. (2010). Involvement of semaphorins and their receptors in neurological diseases. Clin. Exp. Neuroimmunol. 1, 33–45

[B106] Tamagnone L. Artigiani S. Chen H. He Z. Ming G. I. Song H. Chedotal A. Winberg M. L. Goodman C. S. Poo M. Tessier-Lavigne M. Comoglio P. M. (1999). Plexins are a large family of receptors for transmembrane, secreted, and GPI-anchored semaphorins in vertebrates. Cell 99, 71–80 10.1016/S0092-8674(00)80063-X 10520995

[B107] Taniguchi M. Masuda T. Fukaya M. Kataoka H. Mishina M. Yaginuma H. Watanabe M. Shimizu T. (2005). Identification and characterization of a novel member of murine semaphorin family. Genes Cells 10, 785–792 10.1111/j.1365-2443.2005.00877.x 16098142

[B108] Taniguchi M. Masuda T. Mikami Y. Kimura M. Yoshida T. Mishina M. Shimizu T. (2011). Identification and characterization of a novel zebrafish semaphorin. Neurosci. Lett. 488, 215–220 10.1016/j.neulet.2010.11.032 21094219

[B109] Tojima T. Itofusa R. Kamiguchi H. (2010). Asymmetric clathrin-mediated endocytosis drives repulsive growth cone guidance. Neuron 66, 370–377 10.1016/j.neuron.2010.04.007 20471350

[B110] Toyofuku T. Zhang H. Kumanogoh A. Takegahara N. Suto F. Kamei J. Aoki K. Yabuki M. Hori M. Fujisawa H. Kikutani H. (2004). Dual roles of Sema6D in cardiac morphogenesis through region-specific association of its receptor, Plexin-A1, with off-track and vascular endothelial growth factor receptor type 2. Genes Dev. 18, 435–447 10.1101/gad.1167304 14977921 PMC359397

[B111] Usui H. Taniguchi M. Yokomizo T. Shimizu T. (2003). Plexin-A1 and plexin-B1 specifically interact at their cytoplasmic domains. Biochem. Biophys. Res. Commun. 300, 927–931 10.1016/S0006-291X(02)02966-2 12559962

[B112] Vander Kooi C. W. Jusino M. A. Perman B. Neau D. B. Bellamy H. D. Leahy D. J. (2007). Structural basis for ligand and heparin binding to neuropilin B domains. Proc. Natl. Acad. Sci. U.S.A. 104, 6152–6157 10.1073/pnas.0700043104 17405859 PMC1851056

[B113] Waimey K. E. Huang P. H. Chen M. Cheng H. J. (2008). Plexin-A3 and plexin-A4 restrict the migration of sympathetic neurons but not their neural crest precursors. Dev. Biol. 315, 448–458 10.1016/j.ydbio.2008.01.002 18262512 PMC2365924

[B114] Wolman M. A. Liu Y. Tawarayama H. Shoji W. Halloran M. C. (2004). Repulsion and attraction of axons by semaphorin3D are mediated by different neuropilins *in vivo*. J. Neurosci. 24, 8428–8435 10.1523/JNEUROSCI.2349-04.2004 15456815 PMC6729895

[B115] Wolman M. A. Regnery A. M. Becker T. Becker C. G. Halloran M. C. (2007). Semaphorin3D regulates axon axon interactions by modulating levels of L1 cell adhesion molecule. J. Neurosci. 27, 9653–9663 10.1523/JNEUROSCI.1741-07.2007 17804626 PMC6672970

[B116] Wright A. G. Demyanenko G. P. Powell A. Schachner M. Enriquez-Barreto L. Tran T. S. Polleux F. Maness P. F. (2007). Close homolog of L1 and neuropilin 1 mediate guidance of thalamocortical axons at the ventral telencephalon. J. Neurosci. 27, 13667–13679 10.1523/JNEUROSCI.2888-07.2007 18077678 PMC6673613

[B117] Xiang R. H. Hensel C. H. Garcia D. K. Carlson H. C. Kok K. Daly M. C. Kerbacher K. Van den Berg A. Veldhuis P. Buys C. H. Naylor S. L. (1996). Isolation of the human semaphorin III/F gene (SEMA3F) at chromosome 3p21, a region deleted in lung cancer. Genomics 32, 39–48 10.1006/geno.1996.0074 8786119

[B118] Yaron A. Huang P. H. Cheng H. J. Tessier-Lavigne M. (2005). Differential requirement for Plexin-A3 and -A4 in mediating responses of sensory and sympathetic neurons to distinct class 3 semaphorins. Neuron 45, 513–523 10.1016/j.neuron.2005.01.013 15721238

[B119] Yazdani U. Terman J. R. (2006). The semaphorins. Genome Biol. 7, 211 10.1186/gb-2006-7-3-211 16584533 PMC1557745

[B120] Yoshida Y. Han B. Mendelsohn M. Jessell T. M. (2006). PlexinA1 signaling directs the segregation of proprioceptive sensory axons in the developing spinal cord. Neuron 52, 775–788 10.1016/j.neuron.2006.10.032 17145500 PMC1847564

[B121] Yoshida Y. (2012). Semaphorin signaling in vertebrate neural circuit assembly. Front. Mol. Neurosci. 5:71 10.3389/fnmol.2012.00071 22685427 PMC3368236

[B122] Zachary I. C. Frankel P. Evans I. M. Pellet-Many C. (2009). The role of neuropilins in cell signalling. Biochem. Soc. Trans. 37, 1171–1178 10.1042/BST0371171 19909241

[B123] Zhou Y. Gunput R. A. Pasterkamp R. J. (2008). Semaphorin signaling: progress made and promises ahead. Trends Biochem. Sci. 33, 161–170 10.1016/j.tibs.2008.01.006 18374575

[B124] Zimmer G. Schanuel S. M. Burger S. Weth F. Steinecke A. Bolz J. Lent R. (2010). Chondroitin sulfate acts in concert with semaphorin 3A to guide tangential migration of cortical interneurons in the ventral telencephalon. Cereb. Cortex 20, 2411–2422 10.1093/cercor/bhp309 20071458

